# Supplementary material for: Mechanical ventilation as an independent risk factor for mortality in COVID-19-related ARDS: A secondary analysis using propensity score weighting
Source: PLoS One. 2026 Apr 1;21(4):e0344866. doi: 10.1371/journal.pone.0344866 (PMC13042666; doi:10.1371/journal.pone.0344866)
Supplement: Attachment — Submitted filename: PLOS_response_letter_point_by_point.docx [file pone.0344866.s003.docx]

# Response to Reviewers

Manuscript ID: PONE-D-25-62106

Title: Mechanical Ventilation as an Independent Risk Factor for Mortality in COVID-19–Related ARDS: A Secondary Analysis Using Propensity Score Weighting

Dear Academic Editor and Reviewer,

We sincerely thank you for the careful and constructive evaluation of our manuscript. We greatly appreciate the detailed comments, which helped us substantially improve the clarity, methodological transparency, and balance of the paper. We have revised the manuscript accordingly. All changes are reflected in the revised tracked-changes version.

Below we provide a detailed, point-by-point response.

EDITORIAL REQUIREMENTS

Comment: Ensure PLOS ONE style and formatting.

Response:

We revised the manuscript to comply with PLOS ONE formatting requirements, including title page structure, figure citations in text, Supporting Information references, and file naming conventions.

Comment: Refer explicitly to Figure 2 in text.

Response:

We added explicit in-text references to Figure 2 in the Results section where ventilatory longitudinal trajectories are described.

Comment: Supporting Information citations.

Response:

All Supporting Information files are now correctly cited at the end of the manuscript following PLOS ONE guidelines.

Comment: Identifying data in dataset file.

Response:

We removed potentially identifying variables and re-generated the shared dataset with anonymized fields only, in accordance with PLOS ONE data sharing policy and ethics guidance.

REVIEWER COMMENTS – POINT BY POINT

***Comment: Impact is limited by incomplete addition of variables known to affect mortality in ARDS such as steroid use, sedatives, and paralytics.***

Response:

Steroid use has now been added as a covariate in the dataset description, baseline table, and propensity score model specification. Steroid exposure is reported in Table 1 and included in the CBPS/IPTW model. We added a dedicated Discussion paragraph explaining crude imbalance and post-weighting balance.

Sedation exposure and neuromuscular blocker use were not available in the dataset. This is now explicitly stated in Methods and Limitations.

***Comment: Introduction too long and reads like discussion.***

Response:

We substantially shortened and refocused the Introduction, removing extended historical and physiological narrative and focusing on VILI, IMV risk mechanisms, and the study hypothesis.

***Comment: Physiological wording about oxygenation and compliance.***

Response:

Corrected. The Introduction now states ARDS is characterized by hypoxemic respiratory failure and reduced compliance without implying causality.

***Comment: Ventilator parameter table should appear after propensity score analysis.***

Response:

We reordered the Results section so propensity score analysis and ATE estimates appear before longitudinal ventilatory modeling and Table 2.

***Comment: Mechanical power and additional ventilator metrics should be included.***

Response:

Mechanical power has been calculated using a standard simplified formula and added to Methods and Table 2. Tidal volume indexed to predicted body weight (mL/kg PBW) has also been added.

***Comment: Clarify driving pressure clinical relevance.***

Response:

We clarified that median values remained within lung-protective ranges early, while non-survivors showed unfavorable longitudinal trajectories.

***Comment: Prone duration model caveat belongs in limitations.***

Response:

Moved to the Limitations section.

***Comment: Tone down causal claims.***

Response:

We revised Discussion and Conclusions to emphasize association within a causal-inference framework and not proof of direct harm.

***Comment: Steroid effect unclear.***

Response:

We added a Discussion paragraph explaining crude imbalance, post-weighting balance, and residual confounding limits.

***Comment: High-altitude setting should be clarified.***

Response:

Altitude is now explicitly stated in Abstract, Methods, and Discussion.

***Comment: Timing of intubation.***

Response:

Not available in dataset; now explicitly listed as a limitation.

***Comment: ECMO availability.***

Response:

Not available in dataset; now listed as a limitation.

We thank the reviewer again for the insightful comments. We believe the manuscript is now clearer, more rigorous, and better balanced.

Sincerely,

David Rene Rodriguez Lima, MD, PhD

On behalf of all authors
