# Supplementary material for: Mechanical ventilation as an independent risk factor for mortality in COVID-19-related ARDS: A secondary analysis using propensity score weighting
Source: PLoS One. 2026 Apr 1;21(4):e0344866. doi: 10.1371/journal.pone.0344866 (PMC13042666; doi:10.1371/journal.pone.0344866)
Supplement: Attachment — Submitted filename: PLOS_response_letter_point_by_point_auresp_2.docx [file pone.0344866.s004.docx]

**Response to Reviewer #1**

**Manuscript ID:** PONE-D-25-62106R1
**Title:** *Mechanical Ventilation as an Independent Risk Factor for Mortality in COVID-19–Related ARDS: A Secondary Analysis Using Propensity Score Weighting*
**Journal:** PLOS ONE

**Journal requirements**

All references were carefully reviewed to ensure accuracy and completeness. Reference number 28 was corrected due to a formatting error. Importantly, **none of the cited references have been retracted**, which was verified through PubMed and the original journal websites. No changes to the reference list involved the inclusion of retracted articles.

**Reviewer #1 comment**

The reviewer suggested adding the following statement after:

“Variability in prone positioning exposure likely reflects real-world implementation challenges of evidence-based proning protocols, as originally demonstrated in the PROSEVA trial [33], and may relate to operational and clinical heterogeneity during the pandemic period [34,35].”

*“It remains unclear if stricter adherence to proning protocols would have mediated the suspected injury from mechanical ventilation as the mean P/F ratio at diagnosis was <150.”*

**Response**

We agree with the reviewer’s suggestion. **The requested statement has been incorporated into the Discussion section** to acknowledge the uncertainty regarding whether stricter adherence to prone positioning protocols could have mitigated ventilator-associated lung injury, particularly in a cohort with severe hypoxemia at diagnosis (mean PaO₂/FiO₂ <150 mmHg).

All modifications introduced in response to this comment **have been highlighted in red** in the revised manuscript.

Sincerely,

David Rene Rodriguez Lima, MD, PhD

On behalf of all authors
